# Signaling Pathways in Gliomas

**DOI:** 10.3390/genes16050600

**Published:** 2025-05-19

**Authors:** Paulina Stachyra, Ludmiła Grzybowska-Szatkowska

**Affiliations:** 1 II Department of Oncology and Clinical Immunology with Day Chemotherapy, Oncology Centre of the Lublin Region, Jaczewskiego 7, 20-090 Lublin, Poland; 2 Department of Radiotherapy, Medical University of Lublin, Chodźki 7, 20-093 Lublin, Poland; ludmila.grzybowska-szatkowska@umlub.pl

**Keywords:** glioma, cellular pathways, RAS/MAPK/ERK, PI3K/Akt, MGMT, *IDH*, HK2

## Abstract

Changes in cell signaling pathways, which in normal conditions determine the maintenance of cell homeostasis and the correctness of its basic processes, may cause the transformation of a normal cell into a cancer cell. Alterations in cellular metabolism leading to oncogenesis are considered to be a hallmark of cancer cells. Therefore, a thorough understanding of cellular enzymes affecting metabolism and respiration, as well as intracellular pathways connected with them, seems crucial. These changes may be both prognostic and predictive factors, especially in terms of using molecularly targeted therapies. Aberrations in the pathways responsible for cell growth and angiogenesis are considered particularly important in the process of oncogenesis. Gliomas are the most common primary malignant tumors of the brain. The most important molecular disorders determining their particularly malignant nature are aberrations in the pathways responsible for cell growth and angiogenesis, such as the PI3K/Akt or RAS/MAPK/ERK signaling pathway, as well as excessive activity of enzymes, like hexokinases, which play a key role in glycolysis, autophagy, and apoptosis. The multitude of alterations detected in glioma cells, high heterogeneity, and the immunosuppressive environment within the tumor are the main features causing failures in the attempts to implement modern therapies.

## 1. Introduction

Gliomas are the most common primary malignant tumors of the brain [1]. Among adults, the most common tumors are those derived from astrocytes [2]. The global incidence of stellate tumors is 2.98/100,000 and increases rapidly in patients over 40 years of age [2]. The most prevalent cancer in this group is WHO G4 glioblastoma multiforme (GBM), characterized by high malignancy and short overall survival of 13.5 months. The use of radiotherapy and temozolamide in the treatment slightly prolongs the average survival time (15.6 months). The 5-year survival rate is only about 5% [3]. The etiology of brain gliomas remains uncertain, and their pathogenesis is highly complex. Co-occurring alterations in many signaling pathways in charge of cell proliferation, differentiation, and apoptosis, as well as numerous mutations of genes encoding enzymes, are probably responsible [4].

The only modifiable factor that undoubtedly causes an increased risk of developing glioblastoma multiforme is ionizing radiation. Gliomas are more often detected in people who underwent high-dose radiotherapy to the head and neck area in childhood [5], in people suffering from genetic syndromes such as Li-Fraumeni syndrome or Lynch syndrome [6], or in people who have had low-grade glioma in the past [7]. It is indicated that brain tumors are less common in patients suffering from respiratory allergies or atopic diseases [8]. Thus far, no clear evidence has been provided for the relationship between the use of mobile phones and the development of gliomas [9].

Gliomas are classified according to the fifth edition of the World Health Organization (WHO) classification of central nervous system (CNS) tumors, introduced in 2021 [10,11,12,13,14]. Adult diffuse gliomas are divided into groups depending on the mutation status of the *IDH* (isocitrate dehydrogenase) gene and the presence of the 1p/19q codeletion (Table 1) [10].

In the presence of mutations in *IDH1/2,* tumors are classified as *IDH*-mutant; in their absence, they are classified as *IDH*-wildtype. Table 2 presents the main features of gliomas of these two groups.

Cytoreductive surgery remains the basis of glioma treatment, regardless of the degree of malignancy. In the case of WHO G3 and G4 gliomas, it should be supplemented with radiotherapy or chemotherapy alone or a combination of both methods [13]. In approximately 55% of newly diagnosed brain glioma patients, no response is observed to treatment with temozolomide, which is the basic chemotherapeutic drug used in this group of patients. Its use is often associated with the occurrence of side effects, mainly hematological, which disrupt the continuation of treatment [14].

Changes in cell signaling pathways, which in normal conditions determine the maintenance of cell homeostasis and the correctness of its basic processes, may cause the transformation of a normal cell into a cancer cell. Alterations in cellular metabolism leading to oncogenesis are considered to be a hallmark of cancer cells [15]. Therefore, a thorough understanding of cellular enzymes affecting metabolism and respiration, as well as intracellular pathways connected with them, seems crucial. These changes may be both prognostic and predictive factors, especially in terms of using molecularly targeted therapies. Aberrations in the pathways responsible for cell growth and angiogenesis are considered particularly important in the process of oncogenesis.

A literature search was performed manually in Google Scholar, Scopus, and PubMed databases. The review mainly included papers from the last 10 years, as well as papers published earlier.

## 2. Signaling Pathways in Oncogenesis

In the case of gliomas, the most important molecular disorders determining their particularly malignant nature are considered to be aberrations in the pathways responsible for cell growth and angiogenesis, as well as the excessive activity of hexokinases that play a key role in glycolysis, autophagy, and apoptosis [16].

The ability of profound neoangiogenesis is a common feature of all malignant tumors. Gliomas, and especially glioblastoma multiforme, are characterized by an exceptionally developed network of blood vessels that differ from normal vessels. The dense network of blood vessels in gliomas shows structural, functional, and biochemical abnormalities. The vessels have a tortuous course, resembling the glomerulus of the kidney, with blindly ending branches, a reduced number of pericytes and smooth muscles, and large fenestrations of endothelial cells. These disorders determine vascular leakage and unstable blood flow, which in turn leads to tissue hypoxia, development of acidosis, and increased interstitial pressure in the tumor microenvironment [17]. The process of neoangiogenesis in gliomas may have a dual mechanism—dependent on hypoxia or excessive activity of pathways such as the PI3K/Akt signaling pathway (phosphatidyl-inositol 3-kinase-serine/threonine kinase Akt) or RAS/MAPK/ERK (rat sarcoma protooncogene/mitogen-activated protein kinase/extracellular signal-regulated kinase) via mutation or constitutive activation of their components [17]. The state of chronic hypoxia makes the process of anaerobic respiration more favorable for cancer cells. The role of autophagy, *IDH* and *MGMT* mutations, and poly-ADP-ribose polymerases has also been implicated in the pathogenesis of gliomas.

### 2.1. RAS/MAPK/ERK Cellular Pathway

**Figure 1:**
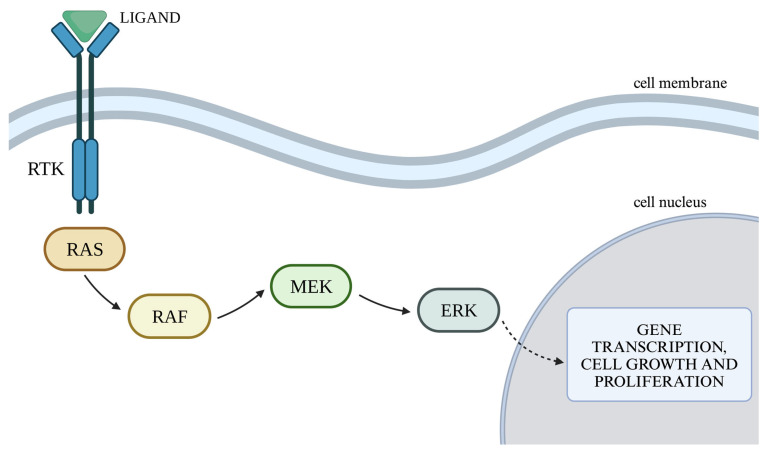
RAS/MAPK/ERK cellular pathway. Legend: EGF—epithelial growth factor; EGFR—epidermal growth factor receptor; ERK—extracellular signal-regulated kinase; MEK—mitogen-activated kinase; PDGFR—platelet-derived growth factor receptor; PDGF—platelet-derived growth factor; RAF—rapidly accelerated fibrosarcoma; RAS—rat sarcoma; RTK—receptor tyrosine kinase; VEGF—vascular endothelial growth factor; VEGFR—vascular endothelial growth factor receptor. Own work using BioRender available at https://www.biorender.com, accessed on 15 May 2025.

The RAS/MAPK/ERK pathway is responsible for signaling from the cell surface to the nucleus, controlling proliferation, differentiation, migration, and survival. Binding of a ligand to a receptor protein with a tyrosine kinase function (RTK) results in the phosphorylation of the RAS (rat sarcoma) protein, which in turn activates the RAF (rapidly accelerated fibrosarcoma) protein with serine–threonine kinase activity. The RAF protein has three isoforms: ARAF, BRAF, and CRAF, with BRAF being the most potent activator of MEK (mitogen-activated kinase). Activated RAF kinase phosphorylates MEK1 and MEK2, which then activate ERK1 and ERK2 (extracellular signal-regulated kinase). Activated ERK kinases transmit a signal to the cell nucleus, leading to increased expression of genes responsible for cell growth and survival (Figure 1) [18,19].

Excessive activation of the BRAF proto-oncogene can result from mutations in the encoding gene or from gene fusion. The most frequently observed BRAF mutation is a missense mutation in codon 600 of exon 15 (V600E), which causes constitutive BRAF kinase activation. This mutation is more frequently observed in lower-grade gliomas (WHO G1-G3) according to the WHO [20]. The BRAF V600E mutation is present in roughly half of pleomorphic xanthoastrocytomas (WHO G2), 20–75% of gangliogliomas (WHO G1), 50% of anaplastic gangliogliomas (WHO G3), and <10% of pilocytic astrocytomas (WHO G1) [20]. BRAF mutations are found in approximately 6% of glioblastomas (WHO G4) [21] but are notably prevalent in the epithelioid subtype (50% of all cases) [20]. Excessive BRAF activation alone is insufficient for gliomagenesis, but animal studies suggested that its combined BRAF and Akt activation may significantly increase the risk of its development [22].

Among all RTKs, the epidermal growth factor receptor (EGFR) and vascular endothelial growth factor receptor (VEGFR), along with their ligands, are considered to play a special role in the pathogenesis of gliomas [15,23]. EGFR can be activated by binding to one of its over 40 ligands, including epidermal growth factor (EGF) and transforming growth factor α (TGF-α) [24]. In glioma cells, EGFR dysregulation may be associated with a mutation of its gene located on chromosome 7p11.2, increased EGFR amount, increased number of gene copies, chromosomal rearrangement, or autocrine activation [25]. EGFR amplification occurs in up to 50% of GBM cases [26], more than half of which carry a mutation in EGFRvIII [27]. The EGFRvIII mutation leads to constitutive activation of the receptor, which does not require its binding to a ligand. It is believed that glioma cells grow more intensively under these conditions [17]. An association has been demonstrated between EGFR amplification and increased invasiveness and between proliferation of glioma cells [24] and resistance to radiotherapy [28]. EGFR overexpression is observed in almost 60% of GBM cases [29]. There is a difference in the frequency of EGFR amplification and EGFR hyperactivity depending on the type of GBM. Such changes are more frequently observed in primary than in secondary tumors [24]. In the absence of EGFR mutations, increased levels of its ligands are often observed, which activates the receptor in an autocrine manner, resulting in autonomous growth of glioma cells [24].

The VEGFR family includes five types of receptors, with VEGFR-1 and VEGFR-2 playing a role in angiogenesis. Binding of VEGF to VEGFR on the tumor blood vessel increases vascular permeability and activates vascular endothelial cell proliferation and migration [30]. It has been shown that increased VEGF concentrations result in the “upregulation” of the VEGFR-2 receptor [31]. The main factor stimulating VEGF is hypoxia. Chronic oxygen deficiency in cells induces the production of hypoxia-inducible factor 1 (HIF1), which is a transcription factor for upregulated production and release of VEGF [15,32]. Increased VEGF activation affects the further activation of pathways responsible for neoangiogenesis in vascular endothelial cells, which is supposed to counteract the effects of hypoxia. This results in increased efficiency of endothelial cell proliferation, growth, migration, and vascular permeability.

Increased release of VEGF by cancer cells, including GBM, is also influenced by elevated levels of EGF. Increased EGF activity in GBM cells is caused by amplification of the receptor gene or its activating mutation [33]. In normoxic conditions, EGF stimulates the activation of two pathways responsible for VEGF induction—the PIK3 pathway and the MAPK/ERK pathway (mitogen-activated protein kinase/extracellular signal-regulated kinase) [33]. Hypoxia has been shown to suppress the PIK3 pathway, reduce PIK3 activity, and cause a switch to the MAPK pathway [34]. A correlation has been indicated between VEGF activity and the degree of histological malignancy of gliomas and a worse prognosis [35]. Meta-analysis of data from previous studies has shown a significant difference in VEGF activity between low-grade gliomas (WHO G1/G2) and high-grade gliomas (WHO G3/G4) [35]. In vivo studies in animal models have shown that VEGF-A is produced by bone marrow cells and stimulates the growth of glioma cells. It has also been shown that VEGF-A produced by non-tumor cells has a stronger effect on tumor growth compared to VEGF-A produced by tumor cells [36].

RAS is one of the most frequently activated oncogenes in human cancers. RAS mutations are mainly observed in codons 12, 13, and 61 [37]. Mutations most frequently concern KRAS (84% of all RAS missense mutations), followed by NRAS (in 12% of cases). HRAS mutations are the rarest and constitute about 4% [38]. The presence of KRAS mutations is supposed to affect the increased activity of VEGF, thus promoting the process of neoangiogenesis of the tumor [39]. The significance of RAS mutations in gliomas has not been explained so far, and there are few publications on this subject in the literature. However, it seems that certain changes in KRAS may determine an increased risk of glioma [38]. A study on an animal model revealed that the KRAS-dependent signaling pathway is essential for the development of GBM in mice, and its inhibition results in tumor cell apoptosis [40]. Even though the role of RAS mutations has been determined in animal models, it is difficult to indicate their association with GBM in humans. Particularly, as the incidence of RAS mutations in gliomas is relatively low—from 1% to 4% [41,42,43]. In a stable state, the RAS protein remains bound to guanosine diphosphate (GDP), whereas binding to guanosine triphosphate (GTP) activates it. The process is regulated by GTPase. Mutations in RAS result in impaired GTPase function, preventing the conversion of GTP bound to RAS protein into GDP, i.e., inactivation of the protein. This leads to its constitutive activity, which results in dysregulation of the proliferation process, promoting oncogenesis [44]. Mutation in NRAS additionally stimulates the PI3K/Akt pathway by directly affecting PI3K [45].

### 2.2. The PI3K/Akt Cellular Pathway and the Autophagy Process

**Figure 2:**
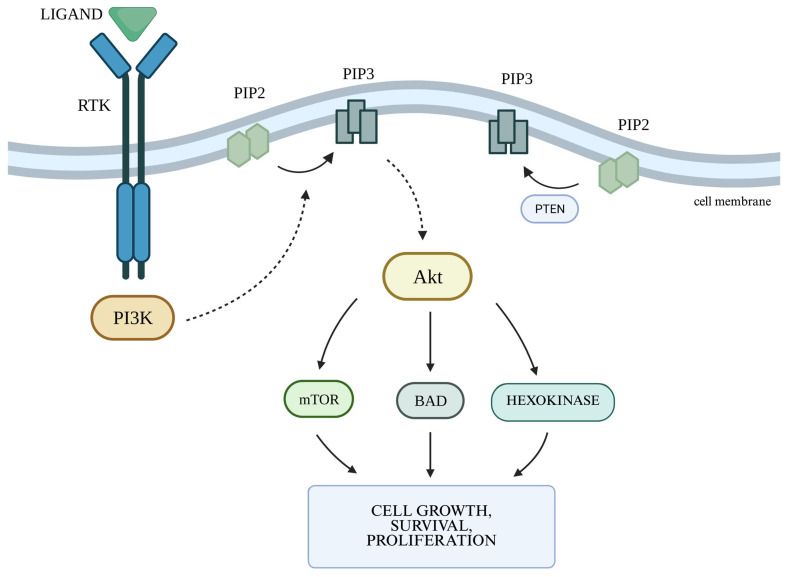
PI3K/Akt pathway. Legend: Akt—serine/threonine kinase; BAD—Bcl-2-associated death promoter; mTOR—serine/threonine kinase mammalian target of rapamycin; PI3K—phosphatidyl-inositol 3-kinase-serine; PIP2—phosphatidylinositol (4,5)-bisphosphate; PIP3—phosphatidylinositol (3,4,5)-trisphosphate; PTEN—phosphatase and tensin homolog; RTK—receptor tyrosine kinase. Own work using BioRender available at https://www.biorender.com, accessed on 15 May 2025.

The PI3K/Akt signaling pathway is one of the main metabolic pathways in which disorders are observed in numerous cancers, including gliomas. They are primarily associated with increased expression of genes whose products participate in the PI3K/Akt pathway [46]. PI3K is regulated mainly by RTKs. The PI3K family includes three classes of kinases, with class I being the most well-known and the most important in the context of oncogenesis and pathogenesis of other diseases [47]. In class I PI3K, we can distinguish two subgroups—IA, which includes isoforms α, β, and δ, and IB, which includes isoform γ [47]. PI3Kα plays a key role in cellular pathways related to angiogenesis, growth, and metabolism, being the isoform most involved in the process of oncogenesis. Ligands activating the pathway by binding to RTKs include growth factors such as EGF and VEGF. Activation of PI3K leads to increased production of phosphatidylinositol (3,4,5)-trisphosphate (PIP3) via phosphorylation of phosphatidylinositol (4,5)-bisphosphate (PIP2). PIP3 is responsible for increased recruitment to the cell membrane and activation of serine/threonine kinase Akt, also known as protein kinase B (PKB). The Akt kinase family consists of three proteins: Akt-1, Akt-2, and Akt-3. Activated Akt kinase regulates, among others, the activity of the proapoptotic Bcl-2 family protein BAD (Bcl-2-associated death promoter) on the outer mitochondrial membrane, as well as the serine/threonine kinase mTOR (serine/threonine kinase mammalian target of rapamycin), thereby regulating the processes of apoptosis, angiogenesis, and proliferation. mTOR kinase functions as a catalytic subunit in two different protein complexes: mTORC1 and mTORC2 (mTOR complexes 1 and2). The PI3K/Akt pathway is controlled by PTEN phosphatase encoded by the suppressor gene PTEN. PTEN phosphatase is responsible for the conversion of PIP3 to PIP2 by catalyzing the removal of the 3′-phosphate group from PIP3, thus acting as a negative regulator for PI3K. When all components of the pathway function properly, they affect the cessation of proliferation and initiate apoptosis (Figure 2) [48].

The relationship between PI3K and autophagy is also important in the process of oncogenesis. Autophagy is a process of intracellular digestion of cytoplasmic macromolecules and organelles in lysosomes. During digestion, biologically active monomers are formed, which are necessary to maintain cellular homeostasis under both optimal and stressful conditions [49]. There are three types of autophagy: microautophagy, macroautophagy, and autophagy associated with chaperones. The best-known process is macroautophagy. Metabolic stress caused by an insufficient supply of oxygen and nutrients is a factor strongly inducing autophagy [50]. Autophagy is a multi-stage process, which consists of initiation, elongation, maturation, and degradation of substrates [49]. The PI3K complex controls the second stage—it participates in the elongation of the phagophore (an isolating membrane that separates part of the cytoplasm) by localizing in its vicinity and recruiting subsequent ATG (autophagy-related genes) proteins, which leads to the elongation and closure of the phagophore in the form of a spherical structure called an autophagosome [51]. The role of autophagy in cancer cells is twofold. On the one hand, it acts as a suppressor on tumor cells by eliminating oncogenic proteins and damaged organelles and maintaining genomic stability [50]. On the other hand, it allows the cell to restore homeostasis by maintaining a constant protein circulation in the cell by reusing compounds released during lysosomal digestion of proteins, protein–lipid complexes, and organelles (i.e., mitochondria, peroxisomes) [50].

However, if cellular stress is not overcome and cellular homeostasis is restored, apoptosis is eventually initiated [50]. The main protein connecting these two processes is beclin 1, which is part of the third class PI3K complex (PI3KC3). This protein, which is crucial for the initiation of autophagy, remains associated with the antiapoptotic protein Bcl-2. Dissociation of beclin 1 from Bcl-2 and its association with the PI3KC3 complex activates autophagy [51]. High Bcl-2 activity has been shown to be associated with resistance to anticancer treatment, while beclin 1 can inhibit cell proliferation in many types of cancer, including glioblastoma multiforme [50]. Moreover, autophagy and the protection it provides to cells under stress conditions may be associated with resistance of cancer cells to anticancer treatment [50].

Hyperactivity of the PI3K/Akt pathway is one of the most frequently observed molecular abnormalities in cancer cells. The literature reports that in up to 80% of GBM cases, one or more abnormalities of the PI3K-related pathway have been observed [52], and PIK3CA (phosphatidylinositol-4,5-bisphosphate 3-kinase catalytic subunit α) mutations are found in approximately 15% of GBM cases [53]. In many cases, gain-of-function mutations in PIK3CA lead to constitutive activation of the PI3K kinase it encodes. Mutation of PIK3CA, located on chromosome 3, is the most common mutation in PI3K. It most frequently affects exon 9 in codons 542 and 545 (E542K and E545K) and exon 20 in codon 1047 (H1047R) [54]. PIK3CA mutations affect the increased activity of signaling pathways that stimulate proliferation and angiogenesis. The occurrence of PIK3CA mutations is associated with a worse prognosis and the risk of rapid relapse of GBM [53]. The most frequently described mutation affecting exon 9 and codon 542 is the substitution of guanine for adenine at position 1624 (c.1624G>A, p.E542K)—a missense gain-of-function mutation [54]. The second most common mutation occurring in gliomas is the PIK3R1 mutation (phosphatidylinositol-4,5-bisphosphate 3-kinase regulatory subunit 1). It affects approximately 10% of GBM cases [53].

Among Akt mutations, the most frequently observed are the activating mutations Akt1 E17K, L52R, and Q79K [55]. The PI3K-dependent pathway can also be activated by EGFR amplification or PTEN mutations, which lead to the loss of PTEN phosphatase function. This mutation is one of the most common in solid tumors, occurring in up to 50–80% of cases [56]. PTEN mutations are observed in 5–40% of gliomas and determine a worse prognosis [57].

The multitude of disorders in cellular processes caused by excessive activation of signaling in the PI3K/Akt pathway seem to be crucial for the oncogenesis process. The PI3K/Akt signaling pathway is associated with the regulation of apoptosis, proliferation, angiogenesis, and glucose metabolism. Its activation leads to the cell’s dependence on a constant high concentration of glucose [46]. Incorrectly activated PI3K/Akt pathway causes cancer cells, including glioma cells, to become resistant to cytotoxic effects, including those associated with anticancer drugs with proapoptotic effects [58].

Interestingly, activation of the PI3K/Akt pathway increases the efficiency of aerobic glycolysis by increasing the activity of hexokinase 2 (HK2)—an enzyme that plays a key role in aerobic glycolysis [59]. Akt can increase HK2 activity by regulating HIF1α activity. HIF1α, a transcription factor accountable for changes in the expression of hypoxia-induced genes, is responsible for the body’s response to reduced oxygen concentrations and eliminating their harmful effects. Increasing its concentration in the cell leads to the intensification of glycolysis [60]. In the HK2 promoter region, there is a site for HIF1 binding. HIF1 binding to HK2 increases enzyme activity [61], while loss of HK2 function reduces tumor vascularization. It may be related to decreased HIF1α activity and pathways associated with, among others, VEGF, which is key to the angiogenesis process, whose activity also seems to be dependent on HK2 activity [62]. Both HK2 and HIF1α have been recognized as independent factors influencing survival [63]. Moreover, a relationship between the activation of the AKT-mTOR pathway caused by PTEN deletion and increased HK2 activity has been demonstrated [64]. It should not be forgotten that this pathway can be stimulated in a non-canonical way by interleukin 6 (IL-6), and therefore activation of this pathway does not have to be the result of a mutation [65].

The role of proapoptotic proteins, which are influenced by Akt, may also be important. Phosphorylation of the BAD protein by Akt prevents its interaction with the proapoptotic protein Bcl-xL, which promotes cell survival [56]. Activation of the proapoptotic protein Bax is also dependent on glucose metabolism and is not possible in the case of increased efficiency of the glycolysis process. It has been proven that HK2 competes with Bax protein for the possibility of binding to the VDAC-1 channel on the mitochondrial membrane [66]. Therefore, the dependence of a cancer cell with an abnormally activated PI3K/Akt pathway on high glucose concentration allows it to avoid apoptosis [56]. This proves the existence of a relationship between PI3K/Akt and aerobic glycolysis.

Disturbances in the PI3K/Akt pathway, which entail the promotion of metabolic processes crucial for the growth and survival of cancer cells, seem to be an excellent target for targeted therapies, also in the case of gliomas. The results of preclinical studies have shown promising results. Based on the knowledge that activation of the PI3K/Akt pathway results in a decrease in the sensitivity of glioma cells to ionizing radiation, the role of PI3K inhibitors in sensitizing glioma cells to radiotherapy by reducing the cell’s ability to repair DNA damage has been proven [67]. Moreover, PI3K inhibition resulted in the restriction of signaling in the PI3K/Akt pathway, which promoted apoptosis and increased the effectiveness of the chemotherapy [68]. However, the tested PI3K inhibitors showed cytostatic rather than cytotoxic effects, even when used in combination with EGFR and mTOR inhibitors [56]. Unfortunately, the results of the clinical studies conducted did not yield such optimistic outcomes. The low efficacy of PI3K/Akt/mTOR pathway inhibitors in the studies conducted so far may be due to the phenomenon of resistance, the basis of which has not yet been explained. Possible causes of this state include secondary mutations, activation of alternative parallel pathways, and amplifications within the further links of the inhibited pathway. Therefore, in order to increase the efficacy of PI3K pathway inhibitors, it may be beneficial to use them together with inhibitors of other pathways. In the case of resistance to mTOR inhibitors, the role of increased MAPK signaling in response to PI3K-RAS activation or excessive activation of RTK resulting from the inhibition of mTOR function is indicated. The process of autophagy may also be important in the development of resistance to PI3K/Akt pathway inhibitors. The use of autophagy by cancer cells as a mechanism guaranteeing survival may result in bypassing cytotoxicity caused by the use of PI3K/Akt pathway inhibitors [48]. Increased autophagy in response to cytostatic drugs is often observed in cancer cells and seems to be associated with the development of multidrug resistance (MDR) [69]. Inhibition of autophagy by PI3KC3 inhibitors resulted in decreased proliferation of renal cancer cells [70] and sensitization to radiotherapy in squamous cell carcinoma of the esophagus [71]. Furthermore, in the non-canonical pathway PI3K/AKT, as was mentioned above, may also be activated by IL-6, which is responsible for the intensification of the inflammatory response and the activation of acute phase proteins, and therefore is essential for the anti-apoptotic signaling cascade, which leads to therapeutic resistance [72].

### 2.3. Other Signaling Pathways in Cancerogenesis

The JAK/STAT3 (Janus kinase/signal transducer activator of transcription protein) signaling pathway participates in many physiological processes, including cell proliferation, immune regulation, and differentiation, and its hyperactivation is generally associated with a poor clinical prognosis [73]. The tyrosine kinase-related receptors, JAK and STAT3, are the three main components within the signaling pathway. Cytokines and growth factors such as interferon, interleukin, EGF, and PDGF transmit signals depending on this pathway [74]. JAK/STAT3 hyperactivation in tumor cells may occur as a result of elevated IL-6 levels in the serum and/or in the tumor microenvironment or loss-of-function mutations affecting negative regulators of STAT3 [75]. Activation of this pathway leads to increased tumorigenic and metastatic ability, the transition of cancer stem cells, and chemoresistance in cancer via enhancing the epithelial–mesenchymal transition [74].

The Notch pathway is a signal transduction mechanism that regulates angiogenesis, cell proliferation, survival, and differentiation. It has been shown to play both an oncogenic and tumor-suppressive role, depending on the specific condition [76]. The Notch receptor and its ligands are transmembrane proteins containing EGF-like repeat sequences [76]. Dysregulation of this pathway promotes epithelial–mesenchymal transition and angiogenesis in malignancies, closely linked to cancer proliferation, invasion, and metastasis [77]. Furthermore, the Notch signaling pathway enhances the cancer invasiveness of glioma cells [78] and is also implicated in conferring chemoresistance to tumor cells [79].

The Sonic Hedgehog pathway is an evolutionarily conserved molecular cascade, primarily associated with central nervous system development during fetal life, and is responsible for signal transmission from the cell membrane to the nucleus. The Sonic Hedgehog signaling networks include extracellular hedgehog ligands, the transmembrane protein receptor called Patched (PTCH), the transmembrane protein called Smoothened (SMO), intermediate transduction molecules, and the downstream molecule Gli [80]. The aberrant activation of the Sonic Hedgehog signaling pathway is caused by mutations in the related genes (ligand-independent signaling) or by the excessive expression of the signaling molecules (ligand-dependent signaling—autocrine or paracrine) and may lead to cancer development [81].

The Wnt/β-catenin, also called the canonical Wnt signaling pathway, is an evolutionarily conserved signaling pathway participating in diverse physiological processes such as proliferation, differentiation, apoptosis, migration, invasion, and tissue homeostasis. The noncanonical Wnt pathway mainly includes the planar cell polarity pathway, Wnt-RAP1 signaling pathway, Wnt-Ror2 signaling pathway, and Wnt-PKA pathway [82]. The aberrant Wnt/β-catenin signaling pathway facilitates cancer stem cell renewal, cell proliferation, and differentiation, playing a crucial role in tumorigenesis and resistance to cancer treatment [83]. Abnormal regulation of the transcription factor β-catenin contributes significantly to tumor initiation and progression [82]. Furthermore, the Wnt/β-catenin signaling pathway orchestrates other cell signaling cascades, such as Notch, Sonic Hedgehog, and PI3K/AKT pathways, which also contribute to cancer development [83].

## 3. Enzymes in Oncogenesis

### 3.1. Isocitrate Dehydrogenase

The isocitrate dehydrogenase (IDH) family consists of three isoforms (IDH1, IDH2, and IDH3) encoded by three different genes with the same names. IDH1 is located in the cytoplasm of the cell and in peroxisomes, while IDH2 and IDH3 are located in the mitochondria [20]. IDH enzymes are involved in the citric acid cycle, also known as the Krebs cycle, which is the final stage of aerobic metabolism. They catalyze the conversion of isocitrate into α-ketoglutarate (α-KG) [84]. The IDH1- and IDH2-catalyzed transformation generates the reduced form of nicotinamide adenine dinucleotide phosphate (NADPH) from nicotinamide adenine dinucleotide phosphate (NADP+), while the IDH3-catalyzed transformation generates the reduced form of nicotinamide adenine dinucleotide (NADH) [20]. Mutations in one of the *IDH* genes are frequently observed in gliomas. They are estimated to occur in 54–100% of diffuse astrocytomas (WHO G2), 66.1% of anaplastic astrocytomas (WHO G3), and 64–93% of oligodendrogliomas (WHO G2 and G3). In the case of GBM (WHO G4), they are much more common in secondary tumors (50–88%) than in primary tumors (5%) [20]. *IDH3* mutations are rarely found in gliomas [85]. *IDH1* mutations occur in gliomas more frequently than *IDH2* mutations. About 90% of *IDH1* mutations are missense mutations consisting of the convergence of arginine to histidine in amino acid residue at position 132 (R132H) of the protein [11]. A similar situation may occur in amino acid residue at position 172 of the protein in the case of *IDH2* mutations [11]. Other frequently described *IDH* mutations include R132C, R132G, R132S, or R132L, but they constitute no more than 8% of all *IDH* mutations detected in gliomas [11,20].

The R132H mutation occurs in approximately 12% of all glioblastomas and in up to 80% of low-grade astrocytomas or GBMs that have progressed from them [86]. Research results indicate that *IDH1/IDH2* mutations, apart from reducing the affinity to isocitrate, also lead to increased production of D-2-hydroxyglutarate (D-2HG) from α-ketoglutarate. D-2HG is an oncometabolite that accumulates in cells. Due to its similarity to α-KG, it inhibits histone demethylase and ten–eleven translocation (TET) methyltransferase, which results in increased DNA methylation. Increased DNA methylation, in turn, inhibits the expression of tumor suppressor genes and disrupts cell differentiation [87].

*IDH* mutations are mostly heterozygous somatic mutations. The presence of a wildtype copy of the gene is necessary to provide a substrate for the mutant enzyme to produce D-2HG. Loss of the wildtype allele, which can occur during tumor progression, also reduces neomorphic activity [84]. It seems that in the last stages of glioma development, the *IDH* mutation is no longer a driver mutation and becomes a passenger mutation when tertiary changes occur in the cell [84]. Moreover, a link has been indicated between the presence of *IDH* mutations and the functioning of the immune system towards tumor cells. Compared with *IDH*-wildtype glioma cells, *IDH*-mutant glioma cells show a lower infiltration of both CD4+ and CD8+ lymphocytes [88]. D-2HG has been shown to exert immunosuppressive effects by inhibiting the proliferation of CD4+ and CD8+ lymphocytes and reducing the migration capacity of T lymphocytes as well as the release of some chemokines [88].

*IDH1* mutation contributes to oncogenesis also by regulating the HIF1α pathway, activating glutaminolysis, and increasing cell sensitivity to glucose deprivation [16]. The mechanism by which *IDH1* mutations affect HIF1α activity remains unclear. In *IDH1*-mutant cells, a decrease in α-KG concentration and an increase in HIF1α production and its target genes VEGF, GLUT1 (glucose transporter 1), and PDK1 (pyruvate dehydrogenase kinase 1), which stimulate the process of neoangiogenesis and cell growth, have been observed. Increased HIF1α production is thought to affect the increased activity of PDKs, which inhibit the activity of pyruvate dehydrogenase by its phosphorylation [89]. Reduced pyruvate dehydrogenase activity results in reduced pyruvate influx into the citric acid cycle [16]. Reduced glutamate concentrations are observed in *IDH1*-mutant cells, which may be due to increased utilization of α-KG to produce D-2HG instead of glutamate.

In GBM cells, elevated glutamine levels have been observed compared to those in healthy brain cells. Glutamine is mostly synthesized from glutamate and ammonia under the influence of glutamine synthetase. Increased glutamine synthetase activity is often observed in GBM. The synthesized glutamine is a source of carbon and nitrogen ions necessary for maintaining cellular homeostasis and cell growth [90].

The mutation status in the *IDH* genes has important prognostic and predictive significance. It has been shown that *IDH*-mutant gliomas have a better prognosis of overall survival compared to *IDH*-wildtype gliomas [84]. One of the studies showed a significant difference in median overall survival in the group of patients with GBM exhibiting the R132H mutation compared to *IDH*-wildtype patients (27.4 months vs. 14 months) [91]. *IDH1/IDH2* mutations lead to increased accumulation of the oncometabolite D-2HG, which affects the disturbances of processes responsible for DNA repair [84]. For this reason, *IDH*-mutant glioma cells are more susceptible to cytostatics and ionizing radiation.

In *IDH*-mutant glioma cells, reduced levels of NADH and NADPH have been observed. They are responsible for protecting the cell against reactive oxygen species, among other functions. NADH and NADPH deficiency may therefore explain the increased sensitivity of *IDH*-mutant cells to ionizing radiation [84].

Increased sensitivity to chemotherapy is probably due to the reduced expression of methylguanine methyltransferase, which is responsible for DNA repair functions. Another important factor is the competitive blocking of α-KG-dependent dioxygenases by D-2HG, which are also involved in DNA damage repair [84]. It has been shown that D-2HG can also alter the functioning of cells directly surrounding the tumor, such as neurons or immune cells. D-2HG has a structure similar to glutamate; therefore, it can activate N-methyl-D-aspartate (NMDA) receptors, causing abnormal discharges in neurons [84]. Studies using D-2HG inhibitors in gliomas have so far yielded conflicting results. They have been shown to inhibit cell growth and prolong survival in mouse models [84].

### 3.2. MGMT Promoter Methylation

MGMT is a key enzyme of the cell nucleus involved in DNA repair. It is responsible for removing the promutagenic alkyl groups from O-6-methylguanine, attached by the use of alkylating drugs, thus protecting the cell from apoptosis. This directly affects the development of resistance to treatment with alkylating chemotherapeutics [92]. MGMT is regulated by methylation of its gene promoter, which leads to a decrease in the activity of this protein and, as a result, increases the sensitivity of cells to the action of alkylating drugs [93].

In the case of gliomas, MGMT promoter methylation is more common in secondary than in primary tumors (73% vs. 43%) [92]. It is detected in about 40% of glioblastomas [93], and its occurrence seems to correlate with the presence of *IDH* mutations. Sources indicate that these changes co-occur in up to 80% of *IDH*-mutant gliomas of lower malignancy grade, according to WHO [93]. In one of the studies, in which 116 glioblastomas and 23 anaplastic astrocytomas were neuropathologically and molecularly examined, a correlation was found between MGMT promoter methylation and *IDH* mutation in 95% of cases [94]. However, in contrast to GBM, which typically loses one copy of chromosome 10 where the gene encoding MGMT is located, lower-grade *IDH*-mutant gliomas usually retain both copies. Thus, MGMT may not be completely silenced, resulting in residual MGMT repair capacity and contributing to resistance to temozolomide therapy [95].

MGMT promoter methylation, much like the *IDH* mutation, is both a prognostic and predictive factor. Patients without MGMT promoter methylation have a poorer prognosis, shorter overall survival (OS), and progression-free survival (PFS) [96]. The presence of MGMT promoter methylation implies a greater benefit from radiotherapy or temozolomide by prolonging OS and PFS [91]. It is, therefore, a predictive factor, and routine assessment of MGMT promoter methylation status to identify patients who may benefit from alkylating therapy seems to be a rational approach.

While MGMT promoter methylation seems to be a beneficial phenomenon, the MGMT expression status may have a completely different meaning. Its increased expression correlates more strongly with OS than MGMT promoter methylation in G3 gliomas. MGMT expression is associated with shorter OS and PFS independently of MGMT promoter methylation [97].

One study analyzed the survival time of patients with recurrent GBM treated with carmustine. A significant effect of carmustine on survival time compared to the control group was demonstrated (266 days vs. 187 days, *p* = 0.02). This effect was particularly pronounced in the group of patients with MGMT promoter methylation (*p* = 0.007) [98]. In the case of resistance to alkylating treatment associated with MGMT, its inhibitors may be used. O6-benzylguanine (O6-BG) is an analog of O-6-methylguanine and inactivates MGMT by transferring an alkyl group. It has the ability to cross the blood–brain barrier and, by inhibiting MGMT, may sensitize cells to the action of temozolomide [99]. Another promising therapy aimed at inhibiting MGMT is RNA interference—a phenomenon consisting of silencing or turning off gene expression by double-stranded RNA that has a structure and sequence similar to the target gene’s DNA sequence. One study on this phenomenon demonstrated that microRNA 198 (miR-198) reduces MGMT levels by inhibiting mRNA translation of this enzyme both in vitro and in vivo. This suggests that the simultaneous use of temozolomide and miR-198 may improve the results of GBM treatment [100].

### 3.3. Poly-ADP-Ribose Polymerases

Poly-ADP-ribose polymerases (PARP) are enzymes participating in processes that are key to cell survival. They are activated during DNA damage, which enables poly-ADP-ribosylation of proteins—a process involving post-translational modification of proteins. These enzymes are essential for implementing repair systems aimed at maintaining genome stability and regulating proliferation and apoptosis processes [101]. In the human genome, 17 genes encoding PARP enzymes have been discovered. The best-known enzyme of this group is PARP1. Reduced PARP1 expression is sometimes observed in cancer cells and leads to reduced genome stability. The task of PARP1 is to repair single-strand DNA damage. In the cell, it is present in the nucleus and mitochondria, where it is a part of the mitochondrial DNA repair complex. The highest PARP1 activity is found in the brain, spleen, and thymus. In normal cells, PARP1 activity is low, but when DNA damage occurs, it increases 10- to 500-fold. The inhibition of its activity promotes the formation of double-strand breaks. Accumulation of such damage can lead to cell death, especially in the case of reduced activity of proteins involved in repairing DNA strand breaks. Changes in the activity of such proteins are observed in various types of cancers and are caused by a range of mutations. The most well-known mutations in this context are those in BRCA1/2—a suppressor gene involved in DNA repair. Pharmacological inhibition of PARP activity turns out to be particularly toxic for cells in which the ability to repair DNA damage is reduced due to the occurrence of a mutation of the suppressor gene. Such cells are almost 1000 times more sensitive to PARP inhibitors than normal cells [101].

The phenomenon of “synthetic lethality” (SL) occurs when mutations in two or more genes coexist and together result in cell death, whereas each mutation alone is non-lethal [101]. When one such gene is damaged, its function is compensated by another gene involved in an alternative pathway to the one in which the mutated gene normally functions. The coexistence of mutations in two such genes results in cellular disorders leading to its death. Due to the multiple rearrangements in the genome of a cancer cell, the loss of function of one of the genes is highly probable. This fact is the basis for the search for drugs that would use the effect of synthetic lethality [100]. Such substances include PARP inhibitors. To date, studies involving these inhibitors have primarily focused on breast and ovarian cancers with BRCA1/2 mutations [101]. PARP inhibitors are approved for the treatment of BRCA-mutated breast cancer, ovarian cancer, prostate cancer, and pancreatic adenocarcinoma.

BRCA mutation is relatively rare in gliomas, but there are a number of other mutations that are associated with effects similar to those related to BRCA mutations. Such mutations are referred to as “BRCAness”. This group includes mutations in *IDH1/2*, *EGFR*, *PTEN,* and the *MYC* proto-oncogene, among others [102]. In normal cells, DNA damage activates repair mechanisms; when repair is not possible, such cells enter the apoptosis path. The accumulation of molecular disorders in glioblastoma multiforme cells determines disorders in the DNA repair mechanisms and the apoptosis process. This contributes to further unaffected cell growth and proliferation, even in the case of accumulated DNA damage. It has been shown that DNA repair mechanisms in GBM cells are constitutively activated, which directly translates into a decrease in the sensitivity of cells to the applied treatment. At the same time, increased PARP1 activity has been reported in glioma cells and is associated with a high degree of malignancy and shorter OS [102]. The use of PARP inhibitors may induce the phenomenon of synthetic lethality and improve the effects of radiotherapy and chemotherapy [102]. Moreover, it has been shown that PARP inhibitors, apart from their synergistic effect with conventional treatment, may also reduce the risk of resistance [101].

### 3.4. Hexokinase 2

The initial glucose metabolism in the cytoplasm of the cell, called glycolysis, is common to both anaerobic and aerobic respiration. In normal tissues and under normoxic conditions, pyruvate produced in glycolysis is converted to acetyl coenzyme A (acetyl-CoA), which then enters the citric acid cycle and oxidative phosphorylation reactions. The process of complete glucose oxidation occurs in mitochondria and results in the production of 32 molecules of adenosine triphosphate (ATP) [103].

Anaerobic respiration prevails in cancer cells, which is a more favorable source of energy acquisition in hypoxic conditions often observed in cancer cells [104]. As a result, glycolysis is used as the main source of ATP, and the resulting pyruvate is converted into lactic acid by lactate fermentation. One of the most important enzymes regulating the glycolysis process are enzymes from the transferase group—hexokinases. In cancer cells, the activity of mitochondrial hexokinases has been shown to increase by up to 200-fold, which is why they have been recognized as the driving force of glycolysis [105,106]. There are four isoforms of hexokinases—I, II, III, and IV, also called glucokinase. HK2 is of particular importance in glycolysis. HK2, encoded by *HK2* present on chromosome 2, catalyzes the reaction of converting glucose transported into the cell by glucose transporters (GLUT) into glucose-6-phosphate [105,107]. It plays an important role in initiating and maintaining the glycolysis process at a high level of efficiency, which is crucial for the growth and proliferation of cancer cells [105,108].

In normal brain cells and low-grade gliomas, HK1 activity and oxidative phosphorylation processes predominate, which indicates a special role of HK2 in the development and growth of malignant gliomas [109].

A relationship between the hyperactivity of the PI3K/Akt/mTOR pathway, often observed in glioma cells, and increased HK2 activity has been demonstrated. Akt activation by factors such as insulin or insulin-like growth factor-1 (IGF-1) results in the activation of mTORC1, which in turn increases the transcription and translation of HIF1α protein. HIF1α then affects the increased expression of *HK2*. In conditions of ischemia and glucose deficiency, the mTORC1-dependent pathway is inhibited by HK2. The combination of HK2 with mTORC1 results in the stimulation of the autophagy process to prevent metabolic deficiencies in the cell. Akt activation affects the increased mitochondrial activity of HK2, increased energy supply, and maintenance of mitochondrial integrity, which results in cell growth and has an anti-apoptotic effect. Upon Akt activation, hexokinase II can translocate to the outer mitochondrial membrane and interact with VDAC, promoting cell survival [110].

**Figure 3:**
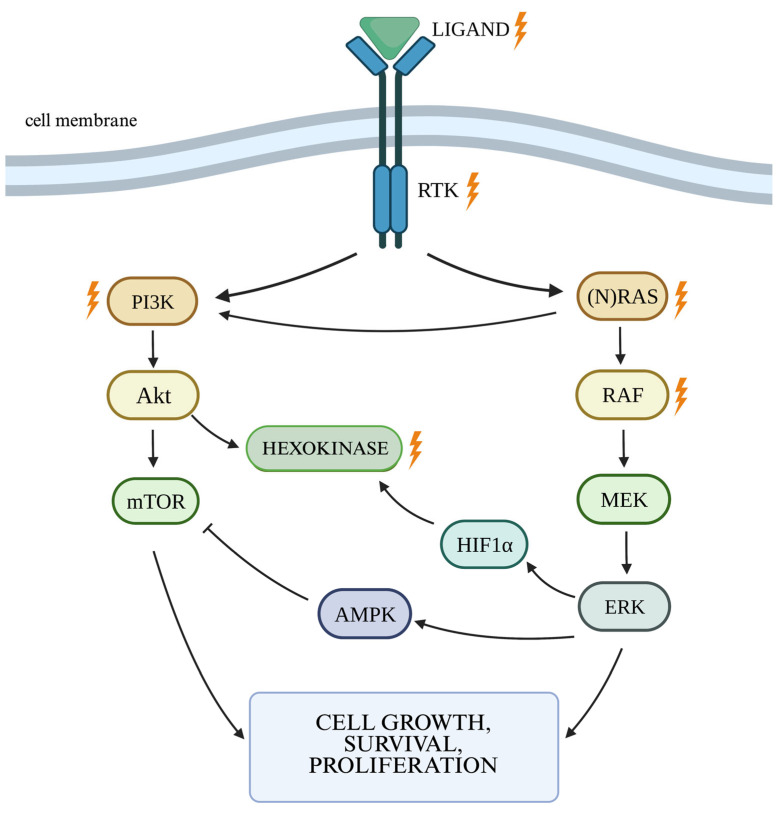
The relationship between PI3K/Akt and RAS/MAPK/ERK pathways and HK2. The bolts indicate the most frequently dysregulated path constituents. Legend: Akt—serine/threonine kinase; AMPK—AMP-activated protein kinase; ERK—extracellular signal-regulated kinase; HIF1α—hypoxia-inducible factor 1α; MEK—mitogen-activated kinase; mTOR—serine/threonine kinase mammalian target of rapamycin; PI3K—phosphatidyl-inositol 3-kinase-serine; RAF—rapidly accelerated fibrosarcoma; RAS—rat sarcoma; RTK—receptor tyrosine kinase. Own work using BioRender available at https://www.biorender.com, accessed on 15 May 2025.

**Figure 4:**
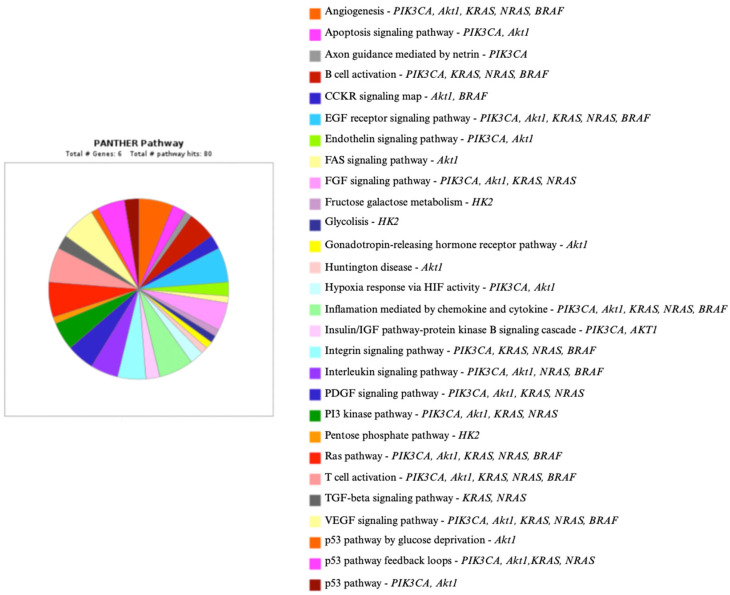
*PIK3CA*, *Akt1*, *KRAS*, *NRAS*, *BRAF*, *HK2,* and their role in cellular pathways [111]. Legend: Akt1—serine/threonine kinase; EGFR—epidermal growth factor receptor; FAS—FAS cell surface death receptor; FGF—fibroblast growth factors; HIF—hypoxia-inducible factor; HK2—hexokinase 2; IGF—insulin-like growth factor; PDGF—platelet-derived growth; p53—protein p53; RAS—rat sarcoma protooncogene; TGF-β—transforming growth factor β; VEGF—vascular endothelial growth factor.

**Figure 5:**
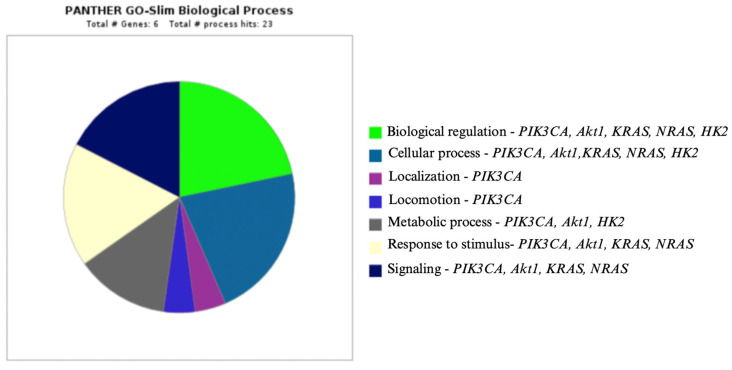
*PIK3CA, Akt1, KRAS, NRAS, BRAF, HK2,* and their role in biological processes [111].

**Figure 6:**
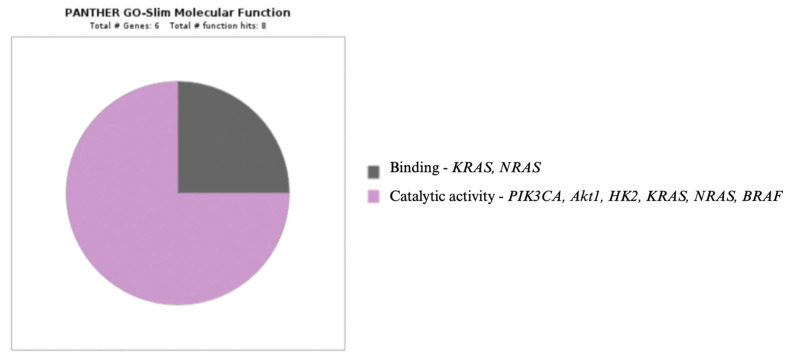
*PIK3CA, Akt1, KRAS, NRAS, BRAF, HK2,* and their molecular functions [111].

The relationships between molecular factors, selected cellular pathways, and hexokinase II are presented in Figure 3, as well as in diagrams generated using the Panther program (Figure 4, Figure 5 and Figure 6) [111].

## 4. Conclusions

Gliomas are a group of tumors characterized by numerous genetic and cellular metabolism disorders that determine their aggressive course. Due to the low effectiveness of treatment with radiotherapy and chemotherapy, researchers are actively seeking aberrations that could serve as potential targets for targeted therapies.

Aberrant activity of pathways such as PI3K/Akt or RAS/MAPK/ERK seems to play a crucial role in the development of gliomas. Due to the chronic hypoxia prevailing in the glial tumor microenvironment, the growth of its cells is strictly dependent on the process of aerobic glycolysis. The high glucose uptake by glioma cells, which determines vigorous growth and promotes survival, indicates the potential role of the glycolysis process as a target for targeted therapies.

Despite initial promising results from preclinical studies, clinical trials have not yielded equally satisfactory outcomes. One of the possible explanations for this situation may be the fact that the tissue samples used in preclinical trials could be characterized by disorders only in the studied pathway, whereas in real-world conditions blocking a single pathway may not be sufficient to achieve an anti-cancer effect. Other possible causes include secondary mutations, activation of alternative parallel pathways, autophagy, and amplifications in the downstream links of the inhibited pathway. Therefore, to increase the efficacy of pathway inhibitors, it may be beneficial to use them together with inhibitors of other pathways.

In the case of HK2 inhibitors, the complex function and high polarity of the HK2 protein may be the main obstacle in finding an effective and selective inhibitor. The problem with the use of HK2 inhibitors may be the fact that this enzyme is also present in normal tissues such as skeletal muscle, cardiac muscle, and adipose tissue. Therefore, systemic use of HK2 inhibitors may entail toxicity due to the lack of specificity. Moreover, cells with elevated HK2 activity often exhibit increased HK1 activity as well, which may contribute to the development of resistance. The presence of the blood–brain barrier also limits the entry of many therapeutic agents into the central nervous system, thus contributing to their lower efficacy.

Therefore, it is crucial to explore and understand the significance of molecular changes occurring in glioma cells. This provides an opportunity for the development of molecularly targeted therapy and better selection of patients for such treatment, which may ultimately lead to a long-awaited breakthrough.

## Figures and Tables

**Table 1:** Division of selected types of gliomas according to the degree of malignancy based on the 5th WHO CNS classification. Own work based on [10].

GLIOMA TYPE	POSSIBLE GRADES OF MALIGNANCY ACCORDING TO WHO
Astrocytoma *IDH*-mutant	2, 3, 4
Oligodendroglioma *IDH*-mutant, 1p/19q codeletion	2, 3
Glioblastoma multiforme *IDH*-wildtype	4

**Table 2:** Division and features of gliomas depending on the presence of *IDH* mutations. Own work based on [11,12].

	*IDH*-WILDTYPE	*IDH*-MUTANT
*IDH* MUTATION	No	Yes
FREQUENCY OF OCCURRENCE	More common in gliomas G4 (>90% GBM)	More common in gliomas G2 and G3
ONSET	Primary	Secondary
LOCATION	Subtentorially	Frontal lobe
AVERAGE AGE OF ONSET	~62 years of age	~44 years of age
PROGNOSIS	Worse	Better

## Abbreviations

The following abbreviations are used in this manuscript:

GBM	glioblastoma multiforme
WHO	World Health Organization
CNS	central nervous system
IDH	isocitrate dehydrogenase
RTK	receptor protein with a tyrosine kinase function
RAS	rat sarcoma
RAF	rapidly accelerated fibrosarcoma
MEK	mitogen-activated kinase
ERK	extracellular signal-regulated kinase
EGFR	epidermal growth factor receptor
EGF	epidermal growth factor
TGF-α	transforming growth factor α
HIF1	hypoxia-inducible factor 1
GDP	guanosine diphosphate
GTP	guanosine triphosphate
PIP3	phosphatidylinositol (3,4,5)-trisphosphate
PIP2	phosphatidylinositol (4,5)-bisphosphate
PKB	protein kinase B
BAD	Bcl-2-associated death promoter
mTOR	serine/threonine kinase mammalian target of rapamycin
mTORC	mTOR complex
ATG	autophagy-related genes
PI3KC3	the third class PI3K complex
PIK3CA	phosphatidylinositol-4,5-bisphosphate 3-kinase catalytic subunit α
PIK3R1	phosphatidylinositol-4,5-bisphosphate 3-kinase regulatory subunit 1
HK2	hexokinase 2
IL-6	interleukin 6
JAK/STAT3	Janus kinase/signal transducer activator of transcription protein
PTCH	patched
SMO	smoothened
NADPH	nicotinamide adenine dinucleotide phosphate
NADH	reduced form of nicotinamide adenine dinucleotide
D-2HG	D-2-hydroxyglutarate
TET	ten–eleven translocation
GLUT1	glucose transporter 1
PDK1	pyruvate dehydrogenase kinase 1
NMDA	N-methyl-D-aspartate
OS	overall survival
PFS	progression-free survival
O6-BG	O6-benzylguanine
miR-198	microRNA 198
PARP	poly-ADP-ribose polymerases
SL	synthetic lethality
ATP	adenosine triphosphate
IGF	insulin-like growth factor-1
